# Surgical Management in a Patient With Complex Uveitic Glaucoma: A Case Report

**DOI:** 10.1097/MD.0000000000001248

**Published:** 2015-08-07

**Authors:** Zhu Huang, Xiao-Yu Wang, Wei Han

**Affiliations:** From the Department of Ophthalmology, The First Affiliated Hospital, College of Medicine, Zhejiang University, Hangzhou, China.

## Abstract

Uveitic glaucoma (UG) is secondary glaucoma, present as a clinical challenge in both diagnosis and management.

We report a case of complex UG, which initially presented as pupillary block and rupture of the anterior lens capsule. We performed cataract extraction with preservation of posterior capsule. Then, the case turned to aphakic malignant glaucoma. We performed anterior vitrectomy with posterior capsule resection in this case. After the second operation, the patient had a satisfactory recovery. Specifically, ultrasonographic biomicroscopy was useful during the diagnosis process and follow-up period in this case.

UG presenting as pupillary block, rupture of the anterior lens capsule, and aqueous misdirection seldom presents in clinical practice. Earlier and more active surgical intervention may be necessary for effective preservation of visual function in complex cases of UG.

## INTRODUCTION

Uveitic glaucoma (UG), first reported by Joseph Beer in 1813, presents a clinical challenge in both diagnosis and management.^1^ The prevalence of glaucoma among uveitis patients ranging from 5% to 20% is greatly influenced by the underlying disease, duration of the disease, and the patient's age.^2^ Clinical manifestations usually comprise uveitis and intraocular pressure (IOP) elevation. The main medical treatment comprises antiglaucoma and immunomodulatory medications. Some severe patients need filtrating surgeries to control IOP, such as goniosurgery, trabeculectomy, deep sclerectomy, and glaucoma drainage device implantation. The common complications include corneal edema, iris synechiae, pupil deformation, cataract, and optic atrophy. However, UG presenting as pupillary block, rupture of the anterior lens capsule, and aqueous misdirection is scarcely observed in clinical practice.

## CASE REPORT

A 44-year-old man presented with pain, redness, and blurred vision of the left eye. Initially, he experienced pain and blurred vision, and visited an ophthalmologist at the local hospital 15 days prior to presenting to our hospital. He was diagnosed with uveitis of the left eye and prescribed intravenous dexamethasone (10 mg in 100 mL, once a day for 10 days) to control the ocular inflammation. On the day before he came to our hospital, the visual acuity of the left eye decreased from 20/30 to counting fingers at 20 cm. Subsequently, the patient elected to visit our eye clinic for alternative treatment. The patient had no family history or systemic disease history, such as rheumatism, tumor, trauma, and others. The treatment and study were approved by the Ethical Review Committee of the First Affiliated Hospital, School of Medicine, Zhejiang University, Hangzhou, China, and the patient permitted his pictures to be published and had signed a consent form.

**FIGURE 1:**
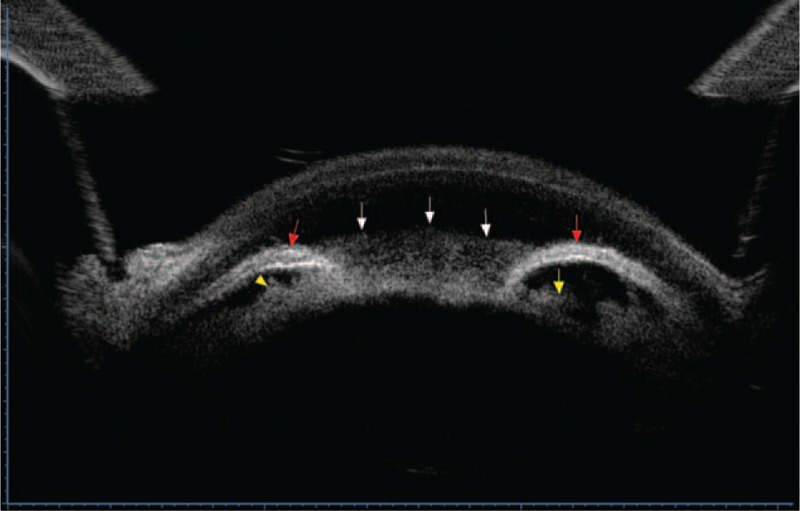
Ultrasonographic biomicroscopy (UBM) image of the left eye shows massive fibrin in the anterior and posterior chambers (white arrow and yellow arrow), shallowing of the anterior chamber, and iris bombe (red arrow). The ciliary body is not well visualized because of the severe inflammatory reaction.

On examination, best-corrected visual acuity was determined at 20/20 in the right eye and count fingers at 10 cm in the left eye. IOP was 12 mm Hg in the right eye and 50 mm Hg in the left eye. Slit-lamp examination revealed apparent ciliary congestion, corneal edema, massive fibrin in the anterior chamber, and shallowing of the anterior chamber in the left eye but normal in the right eye. Anterior chamber angle was shallow with Shaffer grade IV in left eye and wide in right eye. Ultrasonographic biomicroscopy (UBM) of the left eye disclosed massive fibrin in the anterior and posterior chambers, and shallowing of the anterior chamber and iris bombe. Meanwhile, the ciliary body was not clearly visualized because of the severe inflammatory reaction (Figure 1). UBM of the right eye disclosed open anterior chamber angle without inflammation. Ultrasound B scan showed an axial length of approximately 22.13 mm and normal posterior segment anatomy in the left eye. The patient was diagnosed with uveitis of the left eye associated with UG (secondary angle-closure glaucoma). Accordingly, the patient was prescribed the following treatments: 0.3% tobramycin and 0.1% dexamethasone ophthalmic suspension (TobraDex; Alcon Laboratories Inc., Fort Worth, TX) 4 times daily and 0.1% diclofenac sodium eye drops (Shenyang Xingqi Pharmaceutical Co., Ltd., Shenyang, China) 4 times daily, as well as intravenous dexamethasone (10 mg in 100 mL, once a day) to control the ocular inflammation; 1.0% atropine sulfate eye gel (Shenyang Xingqi Pharmaceutical Co., Ltd) twice daily to prevent ciliary spasm; and intravenous mannitol (50 g in 250 mL, once a day), oral methazolamide (25 mg, twice a day), and topical 0.2% brimonidine tartrate and 2% carteolol hydrochloride to lower the IOP.

**FIGURE 2:**
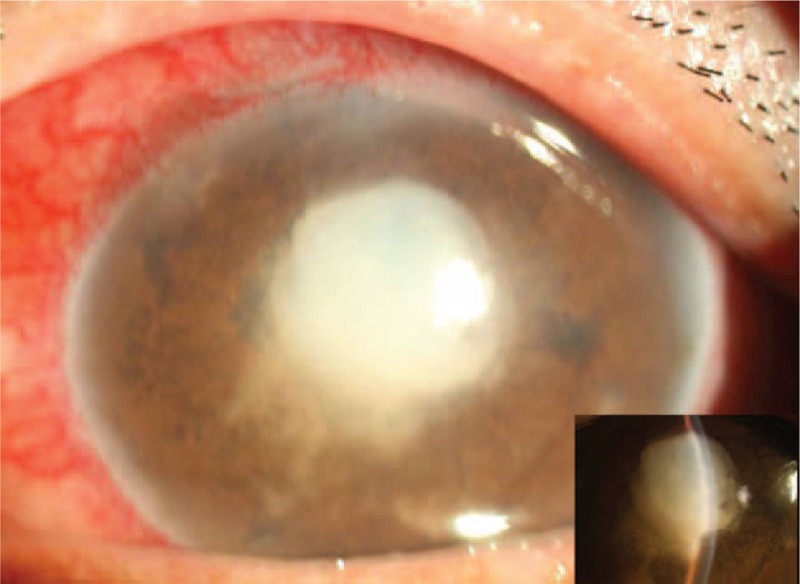
Slit-lamp examination image shows rupture of the anterior lens capsule in the pupil area, migration of the lens cortex into the anterior chamber, and almost complete absence of the anterior chamber.

The medical treatment was ineffective in lowering the IOP. Furthermore, iridotomy with neodymium:YAG laser was performed in the left eye. After laser, the anterior chamber began to deepen, and IOP was 32 mm Hg in the left eye. Unfortunately, 5 days after laser iridotomy, the iridectomy hole was blocked by fibrin. In addition, slit-lamp examination revealed spontaneous rupture of the anterior lens capsule in the pupil area, migration of the lens cortex into the anterior chamber, and nearly absence of the anterior chamber (Figure 2). The anterior capsule rupture and cortical cataract was developed in addition with secondary angle-closure glaucoma, and IOP was further increased. We performed a cortical aspiration on his left eye using a phacoemulsification system (Infiniti phacoemulsification system by Alcon Laboratories, Inc, Fort Worth, TX). Superior and temporal clear corneal incision were made with 3.0 and 1.5 mm knives, respectively. Iris adhesion was separated bluntly. Exudative membranes were picked out with tweezers. A can-opener capsulectomy was performed. Cortical aspiration was made with irrigation/aspiration handle. Although the pupil was difficult to dilate with mydriatic drugs during the operation, the patient underwent cataract extraction (CE) without intraocular lens (IOL) implantation successfully, and the integrity of the posterior capsule was preserved. Aqueous fluid culture for fungi and bacteria was negative. Fibrinous exudation, however, aggravated despite of usage of systemic and topical anti-inflammatory drugs. IOP elevated, and the anterior chamber became shallow again in the third week after the operation.

**FIGURE 3:**
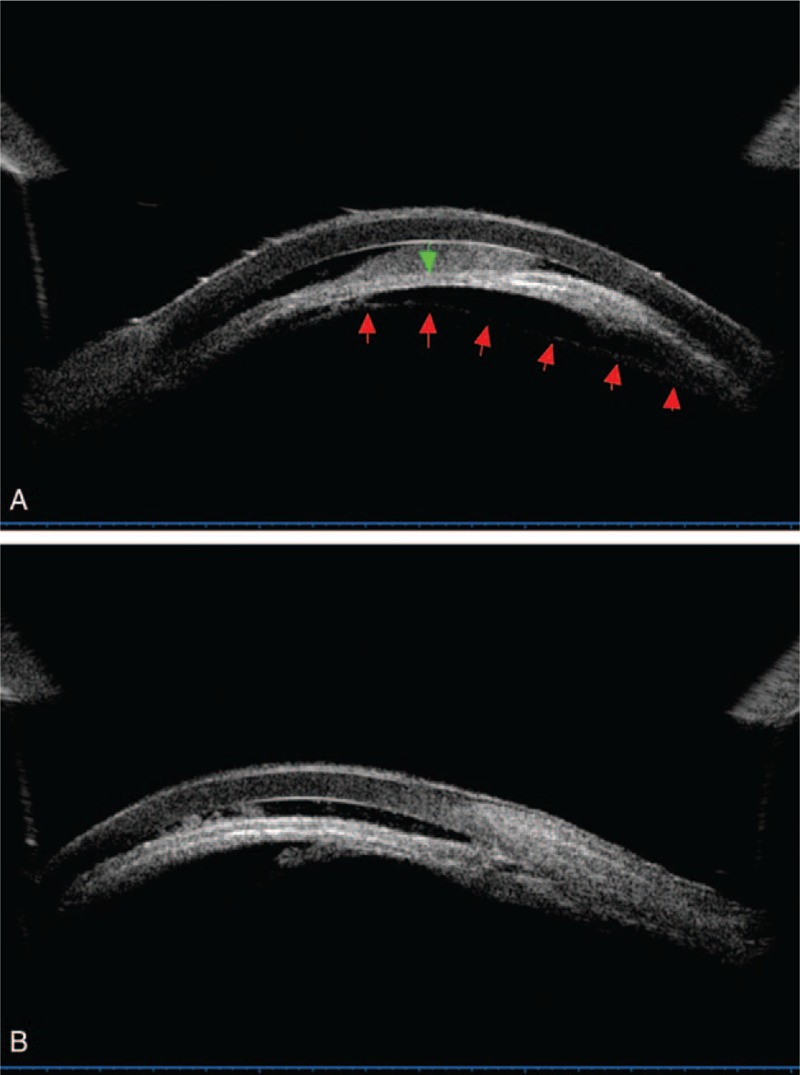
UBM image (A) obtained 25 days after cataract extraction (CE) surgery in the left eye shows fibrin in the anterior chamber, and shallowing of the anterior and posterior chamber (green arrow show shallow central anterior chamber, red arrow show posterior capsule); and image (B) shows forward rotation of the ciliary body.

**FIGURE 4:**
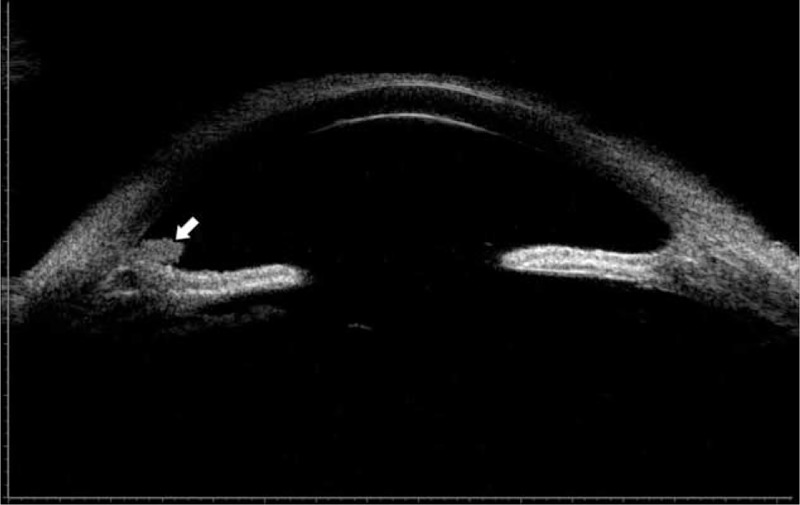
UBM image obtained 1 week after the second surgery comprising anterior vitrectomy and posterior capsule resection shows deepening of the anterior chamber and residual triamcinolone acetonide powder (white arrow) in the anterior chamber angle.

**FIGURE 5:**
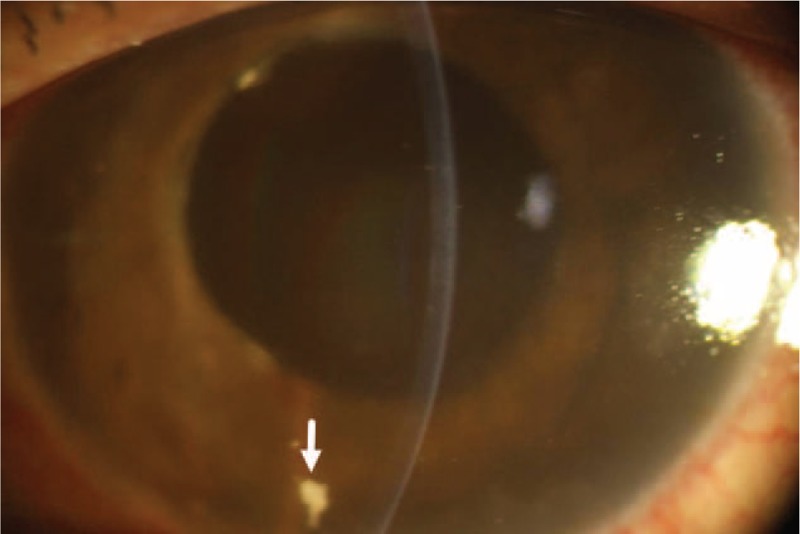
Slit-lamp examination image obtained 1 week after the second surgery comprising anterior vitrectomy and posterior capsule resection shows deepening of the anterior chamber and residual triamcinolone acetonide powder (white arrow) in the anterior chamber angle.

Twenty-five days after the first operation, the patient presented in our eye clinic again. Uncorrected visual acuity had deteriorated to light perception in the left eye. Slit-lamp examination revealed apparent ciliary congestion around the cornea, corneal edema, massive fibrin in the anterior chamber, fibrin membrane across the pupil, and disappearance of the anterior chamber again. UBM of the left eye disclosed fibrin in the anterior chamber, shallowing of the anterior chamber, and forward rotation of the ciliary body (Figure 3). IOP was elevated to 52 mm Hg in the left eye. The patient was diagnosed with left uveitis associated with malignant glaucoma and aphakic eye, and he was admitted to the hospital for reoperation. We performed an anterior vitrectomy with posterior capsule resection and triamcinolone acetonide intravitreal injection on his left eye using an anterior vitrectomy system (CX1000, Bausch & Lomb Microsurgical System, Rochester, NY). Superior temporal scleral incision was made. Removal of exudative membranes, posterior capsulotomy, and anterior vitrectomy were performed. The anterior chamber began to deepen intraoperatively. Then, triamcinolone acetonide (Shanghai General Pharmaceutical Co., Ltd, Shanghai, China) was injected in the vitreous cavity to relieve intraocular inflammation. Vitreous humor culture for fungi and bacteria was negative. Postoperative IOP was 20 mm Hg in the left eye. One week later, his uncorrected visual acuity and best-corrected visual acuity in the left eye were count fingers at 50 cm and 20/60, respectively. Both postoperative UBM and slit-lamp examination showed normal shape of the anterior chamber with residual triamcinolone acetonide powder in the anterior chamber angle (Figures 4 and 5).

One year later, his best-corrected visual acuity was 20/40 in the left eye, and IOP was 18 mm Hg in the right eye and 16 mm Hg in the left.

## DISCUSSION

Approximately 20% of patients with uveitis will eventually develop secondary or so-called UG.^3^ UG can be divided into 2 forms: open angle and angle closure. Acute angle-closure UG is usually associated with angle closure caused by pupillary block secondary to posterior synechia at pupillary margin.^4^ Less commonly, malignant glaucoma coupled with uveitic inflammation and edema result in the angle closure because of the ciliary body rotation.^5^ UG presenting as secondary angle-closure glaucoma, the anterior rupture, along with aphakic malignant glaucoma with posterior capsule was quite rare. The case of aphakic malignant glaucoma with preserved posterior capsule is rarely reported.^6^ To our knowledge, there is no report of aphakic malignant glaucoma with preserved posterior capsule in uveitis patient. What is more—surgical management in such a patient with complex UG has not been described.

The mechanisms of UG are not fully understood. The major etiologic factor might be the increased resistance to aqueous outflow during episodes of intraocular inflammation. In our case, anterior chamber inflammation caused iris posterior synechiae, obstruction of aqueous humor outflow, and iris bombe. Then, angle closure with pupillary block occurred. These findings were clearly visualized on UBM (Figure 1). Of note, there was spontaneous irregular rupture of anterior lens capsule in the central anterior capsule. The reasons of the rupture of anterior capsule include the abnormal fragility of the capsule, the cortical expansion, the iris posterior synechiae, and fibrinous membrane in the anterior chamber. It should be noted that anterior capsule rupture and subcapsular lens opacity may also be produced by laser peripheral iridotomy (LPI).^7,8^ The hole caused by LPI is very round on the peripheral anterior capsule,^7^ which is different from our case.

There are 2 main treatments for UG, medicine and surgery. Drug therapy is important but insufficient in certain high-risk UG cases. Surgery needs to be considered as a vision salvageable procedure and not an end-stage procedure. There are 2 time points of surgery in our case. First, the rupture of the anterior capsular membrane and secondary angle-closure glaucoma could not be controlled by medical drugs or laser therapy. Second, the aphakic malignant glaucoma with uveitis could not be controlled by medical drugs or laser therapy. There are many common surgical options to UG such as laser therapy, trabeculectomy, drainage implants, and cycloablation.^3^ Usually, laser iridotomy is a good treatment to pupillary block. However, when the inflammation of the anterior chamber is active, the obstruction of LPI sites occurs recurrently. Therein, laser iridotomy is not well performed in our case. Similar condition exists after posterior chamber phakic IOL implantation.^9^ In our opinion, surgery that can be performed with more procedures to avoid recurrence of the ocular inflammation, such as removing intraocular inflammatory substances and injection of anti-inflammatory drugs in the vitreous cavity, might be a better choice in this case. Thus, CE was used for eliminating ruptured anterior lens capsule, pupillary block, and cortical expansion cataract. Later, we adopted anterior vitrectomy with posterior capsule resection to treat malignant glaucoma. Finally, the IOP was controlled and the anterior chamber was deepened.

The differential diagnosis of pupillary block and malignant glaucoma involves slit lamp and UBM. Conventional slit-lamp examination is very difficult in the presence of severe anterior chamber exudation associated with elevated IOP. In contrast, UBM with echographic characteristic can visualize the anterior segment even under poor intraocular visibility. In the secondary angle-closure glaucoma, UBM can show iris bombe, shallow peripheral anterior chamber, deep central anterior chamber, and wide ciliary sulcus. In the malignant glaucoma, UBM can reveal forward movement of the iris–lens diaphragm, anterior rotation of the ciliary body, uniformly shallowed anterior chamber, and shallow posterior chamber. In our case, the ciliary body could not be visualized well by UBM owing to the severe intraocular inflammation. However, we could see that anterior chamber and posterior chamber are shallow. As UBM had been available in the different phase of IOP elevation in the present case, an immediate diagnosis would be possible, leading to prompt and effective treatment. After the second operation, UBM confirmed a deep anterior chamber. We thus believed that UBM was useful during the diagnosis process and follow-up period in this case.
